# Genetic Complexity in Recurrent Basal Cell Carcinoma: A MUTYH Variant Case Report

**DOI:** 10.7759/cureus.55677

**Published:** 2024-03-06

**Authors:** Fouad Bouso, Akhaled Zaher

**Affiliations:** 1 Internal Medicine, NewYork-Presbyterian Brooklyn Methodist Hospital, Brooklyn, USA; 2 Internal Medicine, Memorial Sloan Kettering Cancer Center, Brooklyn, USA

**Keywords:** dermatology, gastroenterology, colon cancer, mutyh, basal cell carcinoma

## Abstract

The MYUTH gene plays a critical role in preserving the integrity of the human genome, with mutations being identified in several different cancer diagnoses. It serves its purpose by encoding a DNA glycosylase enzyme responsible for preventing oxidative damage through the excision of adenine that is incorrectly paired with guanine or cytosine. Mutations of the MUTYH gene have been most frequently associated with MUTYH-associated polyposis (MAP) and colorectal cancer. Biallelic mutations of the MUTYH gene are implicated in MAP, and carriers of this mutation have an increased lifetime risk of developing colorectal cancer of 43% to 100%, depending on the appropriate screening and surveillance steps taken. This case describes a patient with recurrent basal cell carcinomas (BCC) and subsequent genetic testing that revealed a pathogenic monoallelic mutation of the MUTYH gene, as well as the interventions that were subsequently performed. It highlights a potentially new patient population that would benefit from early screening to assess the risk of developing colorectal cancers as well as BCC.

## Introduction

Basal cell carcinoma (BCC) is the most prevalent form of skin cancer, typically arising from prolonged exposure to ultraviolet (UV) radiation. It commonly manifests as a slow-growing, non-melanocytic tumor, often found on sun-exposed areas such as the face and neck. While BCC generally remains localized and rarely metastasizes, it can lead to disfigurement if left untreated. Moreover, certain syndromes, like Gorlin syndrome, also known as nevoid BCC syndrome (NBCCS), pose an increased risk of developing multiple BCCs at an early age. Individuals with Gorlin syndrome exhibit various features, including multiple BCC, jaw cysts, skeletal abnormalities, palmar or plantar pits, and, in some cases, medulloblastomas [1]. Additionally, xeroderma pigmentosum, a rare genetic disorder impairing DNA repair mechanisms, heightens susceptibility to BCC and other skin malignancies due to an inability to repair UV-induced DNA damage efficiently. Early detection and management of both sporadic BCC and syndromic forms are crucial to prevent complications and ensure optimal outcomes [2].

We present a complex case of BCC in a 35-year-old female who exhibited unusual genetic variants: a pathogenic MUTYH variant, PTCH1, and SDH variants of undetermined significance. The identification of a pathogenic MUTYH variant in this case is notable, as these mutations are primarily associated with colorectal cancer susceptibility. The presence of this variant underscores the broader implications for the patient's health beyond the dermatological manifestations. As such, the multidisciplinary team approach proved vital in addressing potential gastrointestinal concerns associated with MUTYH mutations, with gastroenterology playing a pivotal role in evaluating the patient's colorectal health [3].

This case highlights the challenges posed by rare genetic variants in BCC and underscores the importance of a collaborative, multidisciplinary approach in managing such cases, especially when these variants have implications for other organ systems.

## Case presentation

**Figure 1:**
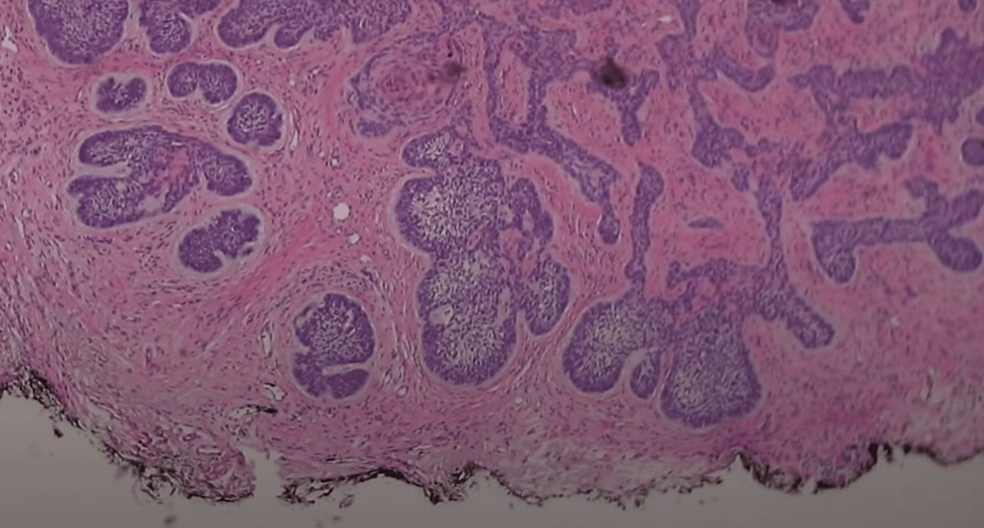
Initial outside shave biopsy demonstrating BCC BCC: basal cell carcinoma

A 35-year-old female with a past medical history of anxiety, GERD, herpes simplex, HPV, and a surgical history remarkable for endocervical adenocarcinoma in situ s/p hysterectomy in 2019 presented in 2021 for further evaluation of BCC on her left nasolabial fold that was confirmed via biopsy (Figure 1).

According to her, the lesion had been present for nearly two years and had begun to scar and bleed for the past several months. Her home medications include alprazolam, bupropion, famotidine, lamotrigine, naproxen, propranolol, quetiapine, and valacyclovir. She has multiple allergies, including penicillin and cats. Her family medical history was also significant for squamous cell cancer and melanoma in her father, stomach cancer in her mother, colorectal cancer in her maternal grandmother, and lung cancer in her maternal grandfather. She is a former smoker of seven years (half a pack per day) and drinks socially.

**Figure 2:**
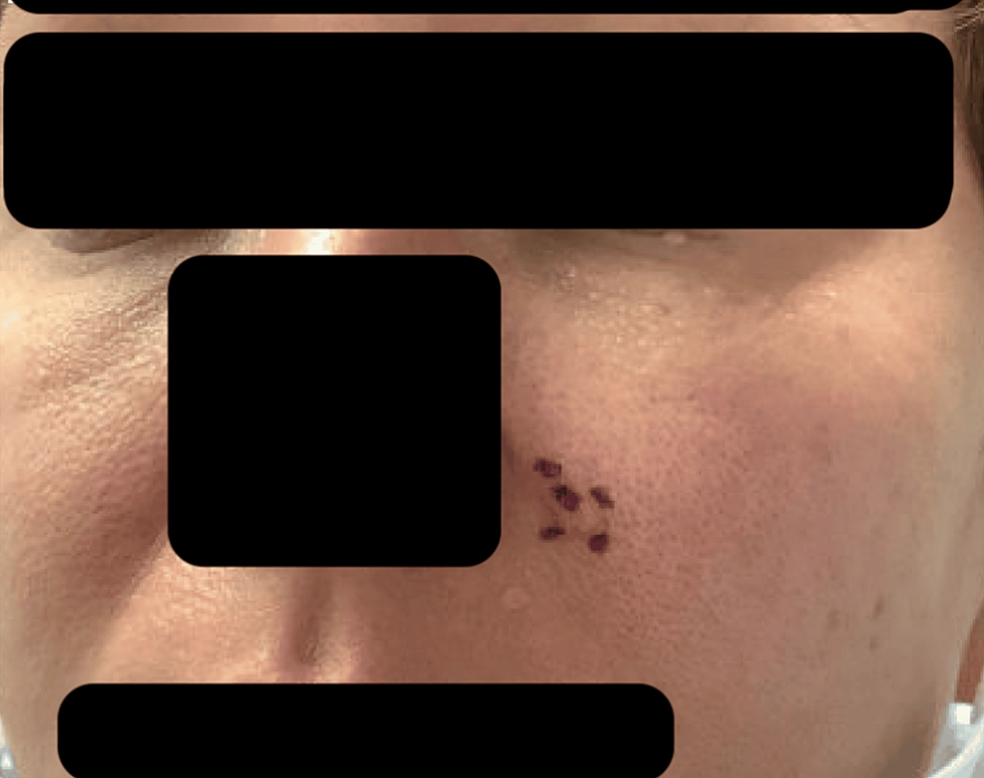
Initial presentation of BCC lesions prior to undergoing first Mohs surgery BCC: basal cell carcinoma

She initially underwent Mohs micrographic surgery of the BCC on her left nasolabial fold in three stages and shaved biopsies of skin-toned papules on the left superior forehead, left infraorbital eyelid, and left upper cutaneous lip (Figure 2).

She subsequently underwent reconstruction and closure of the left cheek wound following the Mohs procedure, which she tolerated. The dermatopathology results of the biopsies showed multiple BCC, at which point it was recommended that she undergo further Mohs surgery for the remaining lesions as well as see a geneticist to assess for possible hereditary involvement. As part of her workup, the patient was referred to a geneticist, where a full pedigree was obtained. Genetic testing revealed a MUTYH pathogenic variant and a PTCH1/SDH variant of undetermined significance.

**Figure 3:**
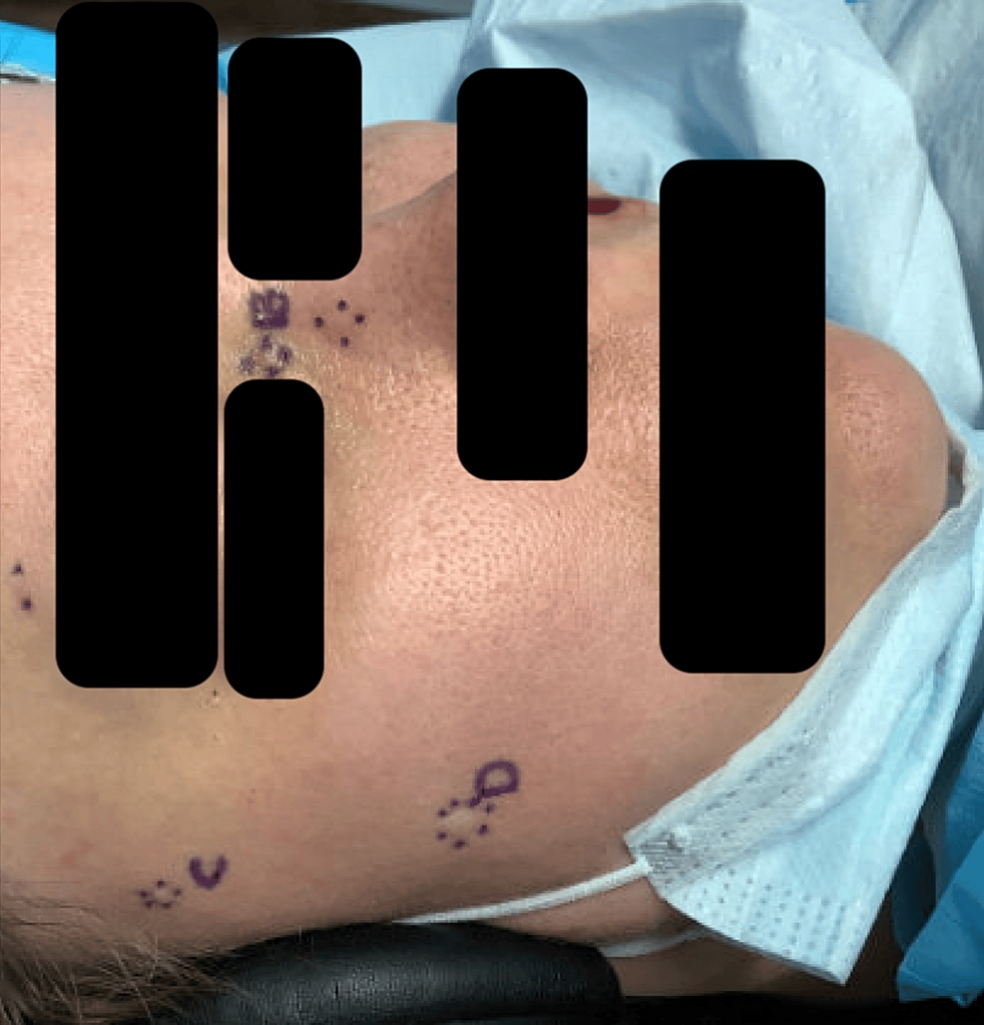
Subsequent facial BCC requiring Mohs surgeries BCC: basal cell carcinoma

She underwent further Mohs micrographic surgery of the lesion on her left upper cutaneous lip in four stages, as well as shaved biopsies of skin-toned papules on her left lower eyelid, right medial canthus, right temple, and right zygomatic cheek. She subsequently underwent further shave biopsies of several other lesions that were present for an undetermined length of time, including the left infraorbital cheek, left medial canthus, right nasal bridge, right medial infraorbital cheek, and right medial canthus (Figure 3).

The pathologies of the areas biopsied repeatedly showed BCC. She once again underwent Mohs surgery, this time on her left lower eyelid, left infraorbital eyelid, left infraorbital cheek, and left medial canthus. Over the next two years, the patient continued to present for repeat biopsies of new lesions and underwent several more Mohs surgeries and reconstructions. Given the complexity of her condition, she was also referred to several specialties for a broader multi-disciplinary approach. Additionally, due to her MUTYH mutation and family history of gastric cancer and colorectal cancer, she was referred to gastroenterology. She was also referred to oncology for systemic care and was started on cemiplimab. She initially tolerated the therapy with no adverse effects; however, after eight cycles of therapy, there was no improvement in her lesions. She then transitioned to vismodegib 150 mg daily. However, two weeks after initiating the new therapy, she was hospitalized when a diffuse erythematous rash developed, and the medication was discontinued. As a result, she was restarted on cemiplimab. Additionally, she began photodynamic therapy (PDT) in January 2023. In February 2023, she was hospitalized for three weeks due to persistent diarrhea, nausea, vomiting, and electrolyte derangement. The infectious workup was negative, and a colonoscopy revealed pan-colitis. This was attributed to the cemiplimab therapy she was undergoing. She underwent treatment with IV steroids with little improvement, so she was subsequently started on infliximab, which yielded improvement in her symptoms. She was then discharged on a prednisone taper, and her cemiplimab was discontinued. Following the completion of her steroid taper, she began to experience recurrent abdominal pain and diarrhea, and she was readmitted to the hospital for two weeks in May 2023. During that time, there was noted to be an elevation in her liver enzymes and inflammatory markers. She underwent a repeat colonoscopy with a biopsy that showed chronic, severely active colitis with ulceration. Upon discharge, she was instructed to follow up with gastroenterology, and she was started on vedolizumab. After failing treatment with cemiplimab and vismodegib, she recently underwent further Mohs surgeries for recurrent BCC lesions. She is currently scheduled to pursue further PDT.

## Discussion

The case of a 35-year-old female with recurrent BCC presents a complex clinical scenario with several facets that have potential implications for clinical practice and research. This discussion explores various aspects of the case, genetic factors, and possible future directions.

One of the variations found during genetic testing was PTCH1, of undetermined significance. Other PTCH1 pathogenic mutations have been found to be associated with NBCCS, which is associated with a variety of other clinical features, including wide-set eyes, intellectual disability, seizures, and keratocystic odontogenic tumors [1]. It presents around the onset of puberty and has an autosomal dominant inheritance pattern [1]. None of these features were present in our patient.

In humans, the MUTYH gene works to repair oxidative damage via DNA glycosylation. It does this by excision of adenine bases in locations where they are wrongly paired with cytosine or guanine [4]. The discovery of a pathogenic variant in the MUTYH gene in this case adds an intriguing genetic dimension to this case. In a 2018 study, 61 patients with frequent recurrences of BCC were enrolled in a study to undergo germline analysis. Nearly 20% of the individuals who participated in the study harbored mutations in DNA repair genes, among which included the MUTYH gene [5]. Although the MUTYH gene is primarily associated with MUTYH-associated polyposis (MAP), a syndrome predisposing an individual to colorectal polyps and cancer [6], it has also been found to be associated with extracolonic cancers, including pancreatic cancer and gastric cancer. One study in patients with pancreatic ductal adenocarcinoma identified the MUTYH gene as a potential target for therapy, where in vitro suppression of AP endonuclease-1 increased apoptosis and subsequently increased susceptibility to chemotherapy [7]. Another study showed that patients with Helicobacter pylori-positive gastric cancer and reduced expression of the MUTYH gene were more likely to have a poorer prognosis [8]. One question that we seek to answer in future studies is whether or not patients who present with recurrent BCC and MUTYH gene mutations warrant earlier or more frequent colorectal cancer screenings.

## Conclusions

The case of this patient with recurrent BCC serves as a striking example of the complexities inherent in managing advanced skin cancers. The identification of a MUTYH gene variant introduces a genetic dimension to the case, encouraging further research into the genetic mechanisms of BCC. This case also underscores the potential to identify a new patient population that may be at risk of internal cancers, especially GI cancers.
